# Diagnostic accuracy of the video otoscope in tympanic membrane perforation

**DOI:** 10.1016/j.bjorl.2023.101336

**Published:** 2023-10-03

**Authors:** Leonardo Resende de Sousa, Guilherme Adam Fraga, Igor Souza Pessoa da Costa, Ana Claudia Ferreira de Almeida, Tyuana Sandim da Silveira Sassi, Luiz Fernando Manzoni Lourençone

**Affiliations:** a Universidade de São Paulo, Faculdade de Odontologia de Bauru, Curso Médico, Bauru, SP, Brazil; b Universidade de São Paulo, Hospital de Reabilitação de Anomalias Craniofaciais, Bauru, SP, Brazil

**Keywords:** Otorhinolaryngology, Otoscopy, Tympanic membrane perforation

## Abstract

**Objective:**

The video otoscope has already proven to be useful for the diagnosis of several pathologies, so the objective of this study was to evaluate the diagnostic accuracy of the video otoscope in cases of tympanic membrane perforation.

**Methods:**

This is a diagnostic accuracy study performed at the hearing health division of a tertiary-level referral hospital. Patients older than 8 years of age who had any symptom that could be related to perforation (otalgia, otorrhea, tinnitus, and/or hypoacusis) were invited to participate in the study. Participants were evaluated by three different diagnostic methods (otomicroscope, conventional otoscope, and video otoscope) performed by three different evaluators in a blind fashion. The microscope was considered the reference standard.

**Results:**

176 patients were evaluated, totaling 352 tympanic membranes. Twenty-seven tympanic membrane perforations were diagnosed by the microscope, a prevalence of 7.7%. The video otoscope showed a sensitivity of 85.2% (95% CI 81.5%‒88.9%), specificity of 98.1% (95% CI 96.7%‒99.5%) and accuracy of 97.1% (95% CI 95.4 %–98.8 %). The conventional otoscope showed a sensitivity of 96.3% (95% CI 94.3–98.3), specificity of 98.8% (95% CI 97.7–99.9) and accuracy of 98.6% (95% CI 97.4–99.8). The Kappa value between the microscope and the video otoscope was 0.8 and between the microscope and the conventional otoscope was 0.9. Regarding the participants' perception, 53.4% (*p* <  0.001) considered the video otoscope as the best method for understanding the tympanic membrane condition presented by them.

**Conclusions:**

The video otoscope showed relevant sensitivity and specificity for clinical practice in the diagnosis of tympanic membrane perforation. Moreover, this is an equipment that can facilitate the patient's understanding of the otologic pathology presented by him/her. In this regard, this method may be important for better patient compliance, requiring further studies to evaluate this hypothesis.

**Level of evidence:**

Is this diagnostic or monitoring test accurate? (Diagnosis)—Level 2 (Individual cross-sectional studies with consistently applied reference standard and blinding).

## Highlights

• 352 tympanic membranes were analyzed.
• 7.7% prevalence of tympanic membrane perforation.
• Sensitivity of 85.2%, specificity of 98.1% and accuracy of 97.1%.
• 53.4% of the participants considered the video otoscope as the best method.

## Introduction

The Tympanic Membrane (TM) is a thin tissue layer that separates the outer ear from the middle ear, playing an important role in transmitting and conducting sound waves to the ear bones.^1^ Because of its location and thickness, the membrane is susceptible to perforation due to inflammatory, traumatic, and neoplastic causes.^2^ Patients with this condition may present the symptoms of otalgia, otorrhea, tinnitus and hypoacusis.^2^, ^3^

Although part of the perforations may heal spontaneously,^4^ the maintenance of an open communication between the external environment and the middle ear predisposes to the occurrence of infections in the middle ear, which may cause serious otologic complications, with conductive hearing impairment and neurological complications.^5^, ^6^ The evolution of this condition is related, among other factors, to the size of the perforation, which influences the possibility of spontaneous healing, the degree of hearing loss and the indication of the need for surgical correction; thus, an accurate evaluation of the membrane during otoscopy is necessary.^7^

In this regard, it is noteworthy that the surgical microscope showed high accuracy in the evaluation of tympanic alterations,^8^ being considered the reference standard as a diagnostic method in this study. Additionally, with the advance of technology in the medical field, several diagnostic devices have evolved over time, and in the field of otorhinolaryngology this was not different. The development of the video otoscope brought the possibility of using the smartphone as an important diagnostic tool in clinical practice, making it easier to obtain TM images and share them with other professionals and with the patients themselves.^9^

Although the video otoscope has been the subject of studies aimed at determining its diagnostic accuracy in other otologic conditions, such as acute otitis media,^10^, ^11^, ^12^ there is no robust evidence in the literature about its accuracy in diagnosing TM perforation.

Thus, the main purpose of this study was to evaluate the diagnostic accuracy of the video otoscope compared to the microscope in cases of TM perforation in patients with otologic complaints seen in the hearing health division of a tertiary-level hospital.

The secondary objectives were to evaluate: the diagnostic accuracy of the conventional otoscope in cases of TM perforation; the agreement between methods regarding the estimated size of the TM perforation; and the participants' preference regarding the method used.

## Methods

This is a diagnostic accuracy study conducted in the hearing health division of a tertiary-level referral hospital in the areas of otology and craniofacial anomaly rehabilitation.

All patients over 8-years of age seen at the outpatient clinic of the study site were considered eligible. The inclusion criteria was the presence of any otologic symptom that indicated otoscopy and that could be related to TM perforation, including otalgia, otorrhea, tinnitus and hypoacusis.

The exclusion criteria were previous history of TM perforation recorded in the participant's medical records and the presence of obstruction in the external ear canal that made it impossible to visualize the TM. It is noteworthy that the cases of obstruction caused by cerumen accumulation were managed by the evaluator responsible for evaluating the conventional otoscope, since the procedure for cleaning the canal is simpler with this method. Once unobstructed, the participant was evaluated by the remaining methods in the sequence.

Informed consent was obtained from the study participants by one of the research team members. In cases of participants between the ages of 8 and 18, in addition to the consent form signed by the child's guardian, an informed consent form was also obtained from the participant.

### Materials

The following materials were used in this study for the TM assessment of all participants:

• OPMI Sensera surgical microscope (Zeiss®)
• Diagnostic Otoscope K100 (HEINE Optotechnik GmbH & Co. KG®)
• WiFi Digital Otoscope R1 (Bebird®):
  - Lens diameter: 4.5 mm
  - Focal length: 1.5–2 cm
  - Pixel: 3 MP
  - Resolution: 1080p
  - Light source: 6 LEDs

### Exposures

After acceptance of participation in the study, all participants were evaluated by three different diagnostic methods (microscopy, conventional otoscopy, and video otoscopy), in a sequential and blind fashion between the three different examiners, which were performed by the otology team of the hospital, composed of otolaryngologists.

The research team defined at the time of the exam which examiner would use each method, so that the examiners did not systematically repeat the method they used. After each evaluation, the physician filled out the electronic form with the participant's otoscopy data and did not communicate the findings either with the patient or with the rest of the research team. Thus, the participant was always submitted to three evaluations performed by three different methods and by three different evaluators.

When describing the otologic physical examination, the evaluator filled in whether the participant had an intact or perforated TM, what the estimated size of the perforation was when present, and reported other otoscopy findings, such as tympanosclerosis, otorrhea, desquamation, etc. The information about the presence or absence of perforation was used to determine the accuracy levels of the methods, as reported below.

After the three otoscopies were done, the findings were shared between the examiners and with the participant, in order to define the management to be followed in each case. In addition, the participants were asked to answer a form with the three questions above:

1) With which diagnostic method did you feel more comfortable (regarding pain, discomfort, examination time)?
2) With which diagnostic method did you feel most uncomfortable (regarding pain, discomfort, examination time)?
3) Which diagnostic method do you consider to have been most useful for a better understanding of your TM's presented condition?

For each question, participants were asked to choose one of the four options listed: microscope, conventional otoscope, video otoscope, or indifferent.

### Sample size

The sample size was calculated based on an estimated sensitivity of the video otoscope in diagnosing cases of TM perforation of 80%, with a maximum margin of error of 15%. These values were indicated from the clinical experience of the hospital specialists. Additionally, a prevalence of 7.8% of TM perforation cases was considered among patients seen at a reference hospital in the area, based on the literature.^2^ Considering, also, the α error as 5% and the β error as 20%, the stipulated sample size was 351 TMs to be evaluated.

Data collection began after project approval by the Ethics in Research Committee of the institution, under protocol n° 4.822.715. The REDCap platform was used for data registration and the project was registered at the beginning of the research in the Brazilian Clinical Trials Registry (ReBEC) platform, under number RBR-3786p33.

### Form of analysis of the results

The data obtained in this study were exported from REDCap and initially submitted to a descriptive statistical analysis. For the analysis of the main objective, McNemar's test for paired data was used, comparing the diagnoses made by conventional methods and by video to the reference standard ‒ microscope. In addition, the Cohen's Kappa Test was performed to assess the agreement between the methods. The Bland-Altman analysis was performed for agreement between the methods regarding the size of the perforation. Finally, for the analysis of the participants' preferences regarding the methods used, the chi-square adherence test was performed.

## Results

**Figure 1:**
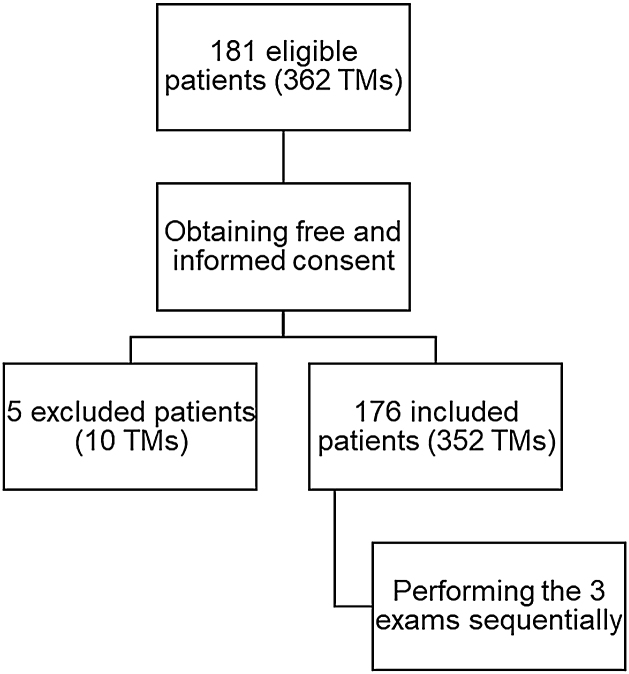
Flowchart of patients eligible for study participation.

According to the sample calculation performed at the beginning of the study, 352 TMs from 176 different patients were evaluated, totaling 1056 otoscopies. Besides these, five patients (10 TMs) were excluded from the sample, since three patients had a record of TM perforation in their medical records, one had bilateral external otitis that made it impossible to visualize the TM, and the last patient had bilateral external ear canal stenosis (Fig. 1).

The sample was composed of 54% (95) of female patients and 46% (81) of male patients. The mean age of the participants was 52.2 (±17.8) years, with the lowest age found being 10 years and the highest being 91 years.

Regarding the complaints reported by the participants, hypoacusis was the most frequent, present in 93.5% (329) of the evaluated ears. In the sequence, tinnitus appeared in 16.4% (58) of the sample, besides otorrhea with 9.4% (33) and otalgia with 4.5% (16).

In the sample evaluated, 27 TM perforations were diagnosed by microscopy, representing 7.7% of prevalence. Of these, 48.1% (13) were in the right ear and 51.9% (14) in the left ear. In these perforation cases, otorrhea appeared as the second most prevalent symptom, with 48.1% (13), behind hypoacusis with 85.2% (23). Besides these, otalgia presented 22.2% (6) and tinnitus 14.8% (4) of prevalence.

In the contingency table (Table 1) are the results of the TM evaluations performed by the video otoscope compared to the reference standard. From the results of the evaluations, it is possible to state that the video otoscope had a sensitivity of 85.2% (95% CI 81.5–88.9), specificity of 98.1% (95% CI 96.7–99.5) and accuracy of 97.1% (95% CI 95.4–98.8) in diagnosing TM perforation. In addition, McNemar's Test was performed to compare the evaluations between the video otoscope and the microscope, showing that there was no statistically significant difference between the otoscopies of the two methods (*p* =  0.527).

**Table 1:** Results of the evaluations performed by the video otoscope compared to the microscope.

	Microscope		
Video Otoscope	Perforated TM	Non-perforated TM	Total
Perforated TM	23	6	29
Non-perforated TM	4	319	323
Total	27	325	352

Table 2 shows the results of the evaluations of the conventional otoscope compared to the reference standard. With these results it was possible to find a sensitivity of 96.3% (95% CI 94.3–98.3), specificity of 98.8% (95% CI 97.7–99.9) and accuracy of 98.6% (95% CI 97.4–99.8) of this method for the diagnosis of TM perforation. McNemar's test also showed no statistically significant difference between the conventional otoscope and the microscope (*p* =  0.180).

**Table 2:** Results of the evaluations performed by the conventional otoscope compared to the microscope.

	Microscope		
Conventional Otoscope	Perforated TM	Non-perforated TM	Total
Perforated TM	26	4	30
Non-perforated TM	1	321	322
Total	27	325	352

The Cohen's Kappa Test was performed to assess the agreement between the methods. The Kappa value for agreement between the microscope and the video otoscope was 0.8 (*p* <  0.001), while the value for agreement between the microscope and the conventional otoscope was 0.9 (*p* < 0.001).

**Figure 2:**
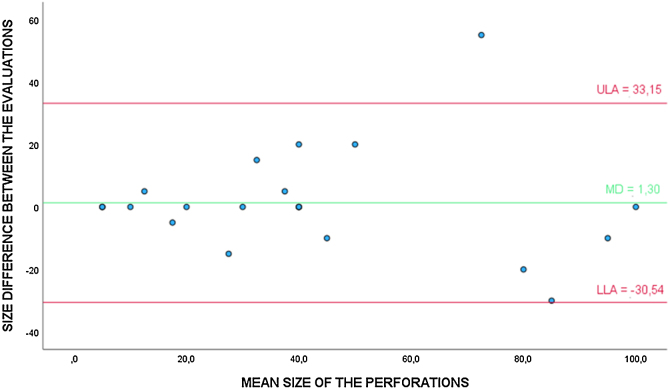
Bland-Altman analysis to evaluate the agreement between the video otoscope and the microscope in the evaluation of the TM perforation size (%). * ULA, Upper Limit of Agreement; MD, Mean of the Differences; LLA, Lower Limit of Agreement.

In addition, the evaluators were asked to estimate the size of the perforation, assigning a value in percentage, being the total perforation considered as 100%. To compare the agreement between the video otoscope and the microscope in the question of the perforation size, the Bland-Altman analysis was performed (Fig. 2) with the cases correctly diagnosed as perforated by the digital method (23). The *t*-test for one group showed that the differences between the evaluations are not statistically different from 0 (*p* =  0.704). Additionally, the linear regression test showed that there was no trend toward overestimation or underestimation of perforation size when evaluated by the video otoscope (*p* =  0.743).

**Figure 3:**
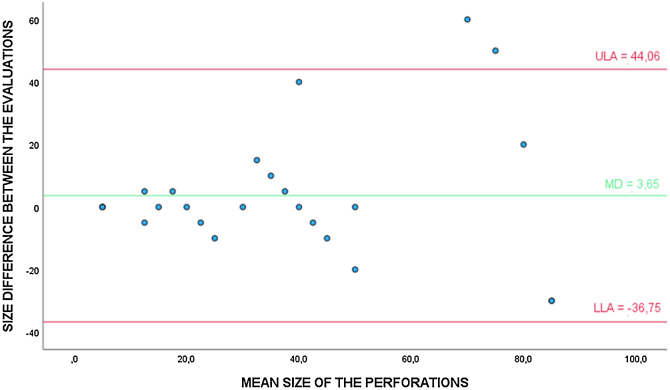
Bland-Altman analysis to evaluate the agreement between the conventional otoscope and the microscope in the evaluation of the TM perforation size (%). * ULA, Upper Limit of Agreement; MD, Mean of the Differences; LLA, Lower Limit of Agreement.

Bland-Altman analysis was also performed to evaluate the agreement between the conventional otoscope and the microscope regarding the size of the TM perforation, in true-positive cases (26), estimated by the evaluators (Fig. 3). The *t*-test also showed that the differences were not significantly different from 0 (*p* =  0.375). In addition, the linear regression test showed no trend towards higher or lower values of this method in relation to the evaluated perforation size (*p* =  0.531).

Regarding the participants' perception of comfort, i.e., the method they classified as the least uncomfortable, the microscope got 26.7% (47) of the votes, the video otoscope got 13.1% (23), and the conventional otoscope got 3.4% (6). However, most patients, 56.8% (100), said there was no difference between the methods regarding comfort, and the Chi-Square test showed a statistically significant difference in this attribution (*p* <  0.001).

Regarding discomfort, i.e., the method that was most uncomfortable during otoscopy, most participants also classified as having no difference between the methods, with 71.6% (126), a statistically significant result (*p* <  0.001). Also in this context, the video otoscope got 14.8% (26) of the votes, followed by the microscope and conventional otoscope with 6.8% (12) each.

As for the method classified by the participants as being the most useful for understanding the otologic condition presented by them, whether normal or pathological, the video otoscope was the most chosen, with 53.4% (94). In addition, 34.1% (60) of people considered that there was no difference between the methods, followed by the microscope with 11.9% (21) and the conventional otoscope with 0.6% (1). The choice of the video otoscope as the best method in this question also showed statistical significance in the Chi-Square test (*p* <  0.001).

## Discussion

The technological advancement of diagnostic methods in medicine has enabled the development of several useful devices in various medical areas. The advent of the video otoscope brought with it the opportunity to capture images and videos of the otologic exam, in a relatively simple process. Moreover, this instrument has also brought the possibility of advancing telemedicine in Otolaryngology, since the images can be shared with specialists at a distance, with the purpose that access to medical diagnosis can be facilitated.^13^

The demand for remote care has intensified even more since the beginning of the pandemic of COVID-19. This has raised the need for discussion of which diagnostic methods are feasible to use remotely. In otology, the video otoscope was highlighted in this context, being analyzed in different ways and in specific pathologies.^14^, ^15^

When used to evaluate the feasibility of the video otoscope in situations where patients, parents, or caregivers themselves obtained the TM images, the results were divergent. One study concluded that the images sent to specialists did not present the necessary quality,^16^ while other researches showed that the files sent to physicians were useful for the diagnosis and follow-up of pathologies, besides presenting adequate patient satisfaction when using the method.^17^, ^18^

In this study, the diagnostic accuracy of the video otoscope was evaluated, in a specific alteration, when used by specialists in otology. In this regard, it can be said that the images obtained have good feasibility for specialists to reach the correct diagnosis. This has positive implications for situations in which a general practitioner obtains the images and sends them to the specialist for consultation regarding the diagnosis. Such a working model has already shown good results in studies where the video otoscope was used as a method to obtain TM images by generalists and sent to specialists at a distance.^19^, ^20^, ^21^

The results found in this study add to those reported in the literature in validating the video otoscope as a reliable method in the diagnosis of various otologic pathologies. One study showed that this method has high sensitivity and specificity rates in the diagnosis of normal or altered membranes.^10^ In addition, the video otoscope was the object of study in studies that evaluated its accuracy in the diagnosis of acute otitis media, also showing good accuracy rates.^11^, ^12^

On the other hand, the observed results differ from that found in another study, which evaluated the diagnostic accuracy of the video otoscope in various pathologies and found an average of 48.6% correct diagnosis of TM perforation.^13^

Extrapolating the clinical context, this tool has also proven useful in teaching otoscopy to undergraduate students. By using this method, students demonstrated greater ability to identify anatomical structures and pathological TM changes, in addition to greater confidence when performing the otologic physical examination.^21^, ^22^ Therefore, this equipment may be important in different contexts and pathologies, reinforcing the need for studies that evaluate its accuracy in certain diseases.

The analysis of agreement between the methods performed with Cohen's Kappa Test showed that both the video otoscope and the conventional otoscope had almost perfect agreement with the microscope, reinforcing the usefulness of the video otoscope in the diagnosis of tympanic membrane perforation.

In this study, based on the Bland-Altman analysis, it was observed that both the video otoscope and the conventional otoscope showed good results in relation to the agreement between the methods regarding the estimated size of the perforation in correctly diagnosed cases. Such statement is based on the fact that both methods presented perforation size averages close to those found by the microscope.

This result is in agreement with the one observed in another study that evaluated the agreement of estimated TM perforation size between a video otoscope connected to a computer and the conventional otoscope. Despite the difference in methodology used in that study, the agreement results were also positive, and the authors recommend that the video otoscope should be used for this purpose whenever possible.^23^

However, it can be seen in the scatter plots (Figure 2, Figure 3) that with the increase in the average perforation size, the difference in relation to the reference standard tends to be greater. It is noteworthy that the estimation of the perforation size is subjective and examiner-dependent, as there are no exact definitions on how to estimate it.

Regarding the participants' preferences as to the method used, it was observed that the video otoscope was chosen by most people as the best method for their understanding of the presented TM condition, whether normal or altered. This fact may be important for better treatment compliance, for example. Studies have shown that for better patient health outcomes, as well as adherence to the proposed treatment, good communication with the healthcare professional is fundamental, and understanding the pathology presented is one of the pillars to achieve this goal.^24^, ^25^

As limitations of this study, we highlight that it was carried out in a single center and, more specifically, in the hearing health division of the hospital, which may be related to the high rate of hypoacusis observed in the sample, both in cases of intact membrane and in cases of TM perforation. Moreover, a non-quantified number of participants had bilateral profound hearing loss, which made it difficult to communicate with them when asking about the preference between the methods in terms of comfort, discomfort, and better understanding of their TM condition. Moreover, the other otoscopy findings were not explored because there was no previous standardization on the recording of the other changes among the evaluators.

## Conclusion

It is possible to conclude that the video otoscope showed high levels of sensitivity, specificity, and accuracy in the diagnosis of tympanic membrane perforation and may be an important diagnostic tool in the context of the pathology evaluated in this study.

In addition, it was observed that both the video otoscope and the conventional otoscope showed satisfactory agreement with the microscope regarding the estimated size of the tympanic membrane perforation.

Finally, the video otoscope proved to be the best diagnostic method for the participants' understanding of the condition of the membrane presented by them, which may be important for the patients' adherence to the proposed treatment, requiring further studies to evaluate this hypothesis.

## Funding

This study was supported by São Paulo Research Foundation (FAPESP), project number 2021/09023-0.

## Conflicts of interest

The authors declare no conflicts of interest.
